# The complete chloroplast genome sequence of *Picea schrenkiana* (Pinaceae)

**DOI:** 10.1080/23802359.2020.1768960

**Published:** 2020-05-27

**Authors:** Jianfang Li, Bei Xu, Qian Yang, Zhan-Lin Liu

**Affiliations:** Key Laboratory of Resource Biology and Biotechnology in Western China (Ministry of Education), College of Life Sciences, Northwest University, Xi’an, China

**Keywords:** *Picea schrenkiana*, chloroplast genome, Pinaceae

## Abstract

*Picea* is a phylogenetically complicate genus with great economic and ecological values. Here, we determined the whole complete chloroplast genome of *Picea schrenkiana* to provide genomic information for phylogenetic analysis of the genus. The plastome of *P. schrenkiana* is 124,060 bp in size and contains 114 genes, including 74 protein-coding genes, 36 tRNA genes, and four rRNA genes. The overall GC content is 38.7%. Unlike the typical plastome with a conserved quadripartite structure, loss of inverted repeat regions is found in the chloroplast genome. The phylogenetic tree shows that monophyly of *P. schrenkiana* is well supported.

*Picea* is a genus of about 35 species of coniferous evergreen trees widely distributed in the North Hemisphere, with great economic and ecological values. The monophyly of the genus has never been doubted, but infrageneric classification and interspecific relationships are quite controversial due to morphological convergence, reticulate evolution, and incomplete lineage sorting (Ran et al. 2006, 2015). Next-generation sequencing technology provides massive genome information for species discrimination and resolution to the complicated phylogenetic issues. Schrenk’s spruce (*Picea schrenkiana*), native to Tianshan mountains, is a major component of subalpine coniferous forest in central Asia. Its population size has sharply decreased for the global climate changes. In the present study, we characterized the complete chloroplast genome of *P. schrenkiana* and analyzed its phylogenetic position. Our results will supply effective data not only for plastome evolution, phylogenetic relationships of *Picea* species, but also for population genetics and conservation works in *P. schrenkiana*.

Genomic DNA was extracted from silica gel dried leaves of *P. schrenkiana* collected in Xinjiang, China (N43.89°, E88.14°). The voucher (2018LIU039) was deposited at the Evolutionary Botany Laboratory (EBL), Northwest University. Data treatments followed our previous study (Li et al. 2019), including data trimming, genome assembly, and annotation. The cp genome was annotated with *Picea crassifolia* (NC 032366) as a reference and has been deposited into GenBank with the accession number of MN871923.

We obtained a larger and more complete plastome sequence of *P. schrenkiana* than those previously reported (Sullivan et al. 2017). The plastome is 124,060 bp long and comprised of 74 protein-coding genes, 36 tRNA genes, and four rRNA genes. The overall GC content is 38.7%. Among the annotated genes, twelve contain a single intron and two (*rps12* and *ycf3*) possess two introns. A typical cp genome of angiosperms has a conserved quadripartite structure with a large and small single copy region (LSC/SSC) separated by a pair of inverted repeat regions (IRs). By contrast, plastomes in Pinaceae species generally exhibit specific repeats (Wu et al. 2011). We examined the size variation of repeat sequences in eleven *Picea* species (Figure 1) and found that two copies of inverted repeats were generally located in *ycf12* and its adjoined genes with the size range of 290–509 bp. *Picea neoveitchii* presented an additional pair of repeat sequences around the *trnI-CAU* and *psbA* gene. No IR-like sequence was detected in both *P. schrenkiana* and *P. jezoensis*. Consequently, Wu et al. (2011) proposed that large repeat sequences other than highly reduced IRs played a critical role in evolution and recombination of gymnosperm plastomes (Sullivan et al. 2017).

**Figure 1.**
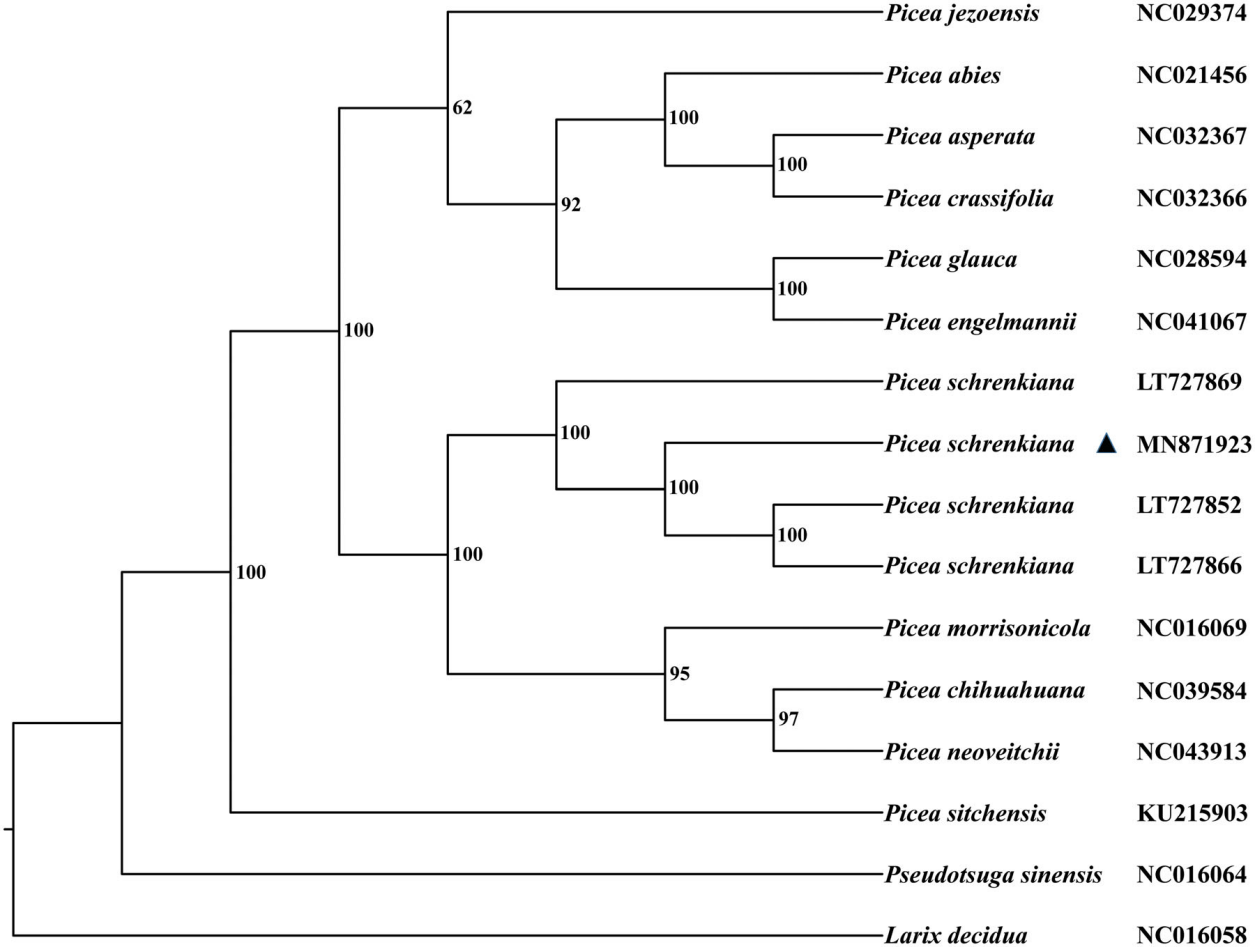
The phylogenetic tree of eleven *Picea* species constructed by complete chloroplast genomes. The bootstrap values labeled beside the branches were based on 1000 replicates.

After alignment with MAFFT v7.310 (Katoh and Standley 2016), 14 plastomes of *Picea* were used to construct the Maximum-Likelihood (ML) tree with *Pseudotsuga sinensis* and *Larix decidua* as the outgroup. The procedure of tree construction was referred to Peng et al. (2019). Our result shows that *P. schrenkiana* is clustered together with its congeners and interspecific relationships of *Picea* could be also clearly clarified in the ML tree (Figure 1).

## Data Availability

The data that support the findings of this study are available on request from the corresponding author, or in NCBI at https://www.ncbi.nlm.nih.gov.
